# Are Marbling and the Prediction of Beef Eating Quality Affected by Different Grading Sites?

**DOI:** 10.3389/fvets.2021.611153

**Published:** 2021-03-29

**Authors:** Jingjing Liu, Grzegorz Pogorzelski, Alix Neveu, Isabelle Legrand, David Pethick, Marie-Pierre Ellies-Oury, Jean-François Hocquette

**Affiliations:** ^1^INRAE, Université Clermont Auvergne, VetAgro Sup, UMR1213, Recherches sur les Herbivores, Clermont-Ferrand, France; ^2^Department of Technique and Food Development, Faculty of Human Nutrition and Consumer Sciences, Warsaw University of Life Sciences (WULS-SGGW), Warsaw, Poland; ^3^École Nationale Supérieure Agronomique de Toulouse, Toulouse, France; ^4^Institut de l'Elevage, Service Qualité des Carcasses et des Viandes, MRA-NA, Limoges, France; ^5^School of Veterinary and Life Sciences, Murdoch University, Perth, WA, Australia; ^6^Bordeaux Sciences Agro, Bordeaux, France

**Keywords:** marbling, meat color, fat color, grading site, beef carcass, Meat Standards Australia (MSA) grading scheme

## Abstract

For the European abattoirs, the preferred carcass grading site is at the fifth rib, and cutting at the tenth rib as in Australia could lead to a lower economic value of the carcass. Therefore, the objective of this study was to compare the grading scores of marbling and the meat and fat color on *Musculus longissimus thoracis et lumborum* (LTL) at the fifth and the tenth thoracic vertebrae. The consequences on the prediction of beef eating quality using the Meat Standards Australia (MSA) grading scheme were also evaluated for cull cows, which produce the majority of beef consumed in France. Carcasses from 208 French cattle, mainly Limousine cows, were graded according to the Australian Beef Carcase Chiller Assessment System (ABCAS) used for the implementation of the MSA system. The results indicate that there was no significant difference in the marbling score, between the fifth and the tenth ribs and hence in the MSA index and in the Global Quality [meat quality (MQ4)] scores calculated from marbling values from either the fifth rib or the tenth rib. However, the meat color at the tenth rib was significantly darker than that at the fifth rib (*p* < 0.01), and the fat color at the tenth rib was significantly yellower than that at the fifth rib (*p* < 0.001). The results of this study suggest that the grading of marbling can be conducted on *M*. LTL at the fifth thoracic vertebrae for routine use of the MSA system in France and, more generally, in Europe. However, further investigation and adjustment would be needed for other critical MSA scores (such as rib fat thickness) while respecting the European carcass quartering practices.

## Introduction

A regular decline in beef consumption has become a big challenge for the European beef industry (1). France, the largest beef producer in Europe, has also experienced a decreasing trend in beef consumption (2). However, despite the declining consumption, FranceAgriMer (3) reported that the household interest in premium beef was growing. Ellies-Oury et al. (4) also demonstrated that an eating quality guarantee scheme would be of interest to French consumers.

To date, the most advanced beef grading scheme is probably the Meat Standards Australia (MSA) system, which has been known as the most well-established beef eating quality guarantee system (1). The aim of the MSA grading system is to ensure that, when consumers purchase a cut of beef, it will have the eating quality promised by the MSA label when it is cooked according to the recommended method (5). The meat quality (MQ4) score was developed to rank the potential eating quality of individual muscle cuts, and the MSA index is used to assess the average eating quality across the whole beef carcass (6).

In France, where 61% of the beef consumption originates from cull cows (7), the “Label Rouge” quality sign has been used to ensure the eating quality of cuts for the consumer, especially for beef produced from the late-maturing breeds (such as Charolais or Limousine breeds). Recently within the framework of the French national food conference (8), the French meat sector represented by Interbev decided to increase the proportion of labeled beef using the “Label Rouge” and other quality signs. The purpose is to assist consumers to make purchase decisions with reliable label guidance (4), which is consistent with the aim of the MSA grading scheme. Indeed, the latter is a reliable description system of eating quality that could form a basis for retail pricing and generate product confidence for consumers (9).

Subsequently, several research efforts have been conducted in Europe to disseminate the MSA methodology as a reference (10–12) under the auspices of the United Nations Economic Commission for Europe (UNECE) (13). Being one of the critical steps in the MSA system, carcass grading parameters contribute to the basis of the beef palatability prediction model (14). Carcass grading is performed under the guidance of the Australian Beef Carcase Chiller Assessment System (ABCAS). According to ABCAS, marbling, meat color, and fat color can be assessed on *Musculus longissimus thoracis et lumborum* (LTL) at any ribbing site from the fifth to the thirteenth rib (15) for cattle routinely slaughtered in Australia, most of which are young steers and heifers. This type of research is still lacking with regard to old cows and/or mainly late-maturing breeds for any potential application of the MSA in the European countries, particularly France.

In general, the most common grading site used by abattoirs in Australia is from the tenth rib to the twelfth rib (16). In contrast, in the European system, quarter carcasses are sometimes sold to the market without further processing. Consequently, the quartering site in most cases is at the fifth thoracic vertebrae, and so the cutting at the tenth thoracic vertebrae would often negatively influence the economic value of the hindquarters.

Thus, the present study aimed to investigate any difference in LT marbling scores, which were collected according to the ABCAS procedure between the fifth and the tenth thoracic vertebrae along with the meat color and the fat color. The potential impact of the different grading sites on the prediction of beef eating quality (through MQ4 scores for each cut and the MSA index for the whole carcass) was also examined. To complete the previous research with young animals from early-maturing breeds (15), this study was mainly conducted with cull cows (which are the major source of beef in France and, thus, have a strong economic significance) from a famous and highly distributed late-maturing breed (the Limousine), producing beef that were already commercialized with the “Label Rouge” quality sign.

## Materials and Methods

### Animals and Experimental Design

The data used in this study were from 208 carcasses (including 157 Limousine breeds) provided by a commercial slaughterhouse in Limoges, France. All carcasses were assessed 24 h after *post-mortem*. Carcasses were graded by using a single MSA-accredited grader for at least 20 min after the cutting to allow the meat to bloom. Assessments, primarily at the tenth rib site and the secondarily at the fifth rib site, were carried out by using the same grader, who is highly experienced with carcass grading according to ABCAS specifications. He is also an official grading trainer recognized by AUS-MEAT. The conditions of the carcass and the environment, including ribbing height and angle and grading practices, such as light angle, are consistent for all assessments conducted at the two rib sites. For the experiment, the AUS-MEAT marbling, MSA marbling, meat color, and fat color were assessed at the fifth and the tenth rib of the same half carcasses (16). The basic information of the current samples is presented in Table 1. European conformation and fat scores were both converted into a continuous 15-point scale as described in the previous study (17).

**Table 1:** Number, mean, SD, minimum, and maximum values for the basic carcass traits.

	***n***	**Mean**	**SD**	**Min**	**Max**
Age (days)	204	3,458	1,835	228	7,422
Carcass weight (kg)	204	356.5	95.8	126.8	729.7
Ultimate pH	198	5.7	0.16	5.3	6.3
Ossification score	185	480	160.8	100	590
European conformation score^a^	204	8.7 (*R =*)	3.1	1	13
European fat score^b^	204	7.0 (3-)	1.00	3	9
Hump height (cm)	204	6.9	2.5	3.5	18

a *European conformation score converted from P (–/=/+), O (–/=/+), R (–/=/+), U (–/=/+), and E (–/=/+) to 1–15*;

b *European fat score converted from 1 (–/=/+), 2 (–/=/+), 3 (–/=/+), 4 (–/=/+), and 5 (–/=/+) to 1–15*.

### Data Collection

All assessments were conducted by following the specifications of the ABCAS and the AUS-MEAT Reference Standards, which include the ossification score at the carcass level, as well as the assessment of marbling, meat color, and fat color on *M*. LTL.

The AUS-MEAT marbling score reflects the number of marbling, ranging from 0 to 9 in increments of one. The MSA marbling score is used to provide a more precise marbling scale in comparison to the AUS-MEAT and is based on the United States Department of Agriculture system (18): it provides scores ranging from 100 to 1,190 in increments of 10. The MSA marbling score indicates not only the amount of marbling but also the size, fineness, and distribution of fat inclusions in muscles (19).

The fat and meat colors are scored according to the AUS-MEAT scale. Fat color is from 0 to 9. Meat color is from 1A, 1B, 1C, and then from 2 to 7 (which was converted into the following scale in this study: 1, 1.33, 1.66, 2 to 7).

### Prediction of MQ4 Scores and the MSA Index

The MSA grading scheme allows the calculation or the prediction of a single palatability or MQ4 score that describes the complete eating experience of a consumer. It is defined as the combination of four sensory traits, namely tenderness, juiciness, flavor liking, and overall liking (20).

The MSA prediction model allows the prediction of MQ4 scores of individual muscles from the carcass for a range of aging time, hanging method, or cooking techniques using a multiple regression approach. The parameters used to predict MQ4 include, among others, animal sex, carcass weight, hanging technique, hump height, ossification score, marbling score, rib fat depth, ultimate pH, and days aged (20). In this study, MQ4 scores were predicted for three cuts [called CUB045 [*M. longissimus thoracis* (LT)], STA045, and STP045 (*M*. LTL) in the MSA grading scheme] that represent different portions of *M*. LTL, where the marbling scores have been recorded. All carcasses were assumed to be Achilles hung (the most common method) and the cuts were assumed to be aged for 10 days and grilled. The prediction of MQ4 scores was made twice for each cut with the same inputs except for marbling scores, which were recorded either at the fifth rib or at the tenth rib, the predictions were made using the SP2009 version of the MSA model.

Then, the MSA index was predicted at the carcass level using the same inputs. The MSA index corresponds, by definition, to the sum of the predicted MQ4 scores of all MSA cuts, the weight of each was calculated as the percentage of the total weight of the MSA cuts in the carcass. To enable a standardized index reporting, inputs are standardized for the calculation of the MSA index: all carcasses are assumed to be Achilles hung (the most common method), and all cuts are assumed to be aged for 5 days and cooked according to the most common cooking method for each cut (6). In this study, the MSA index was predicted twice with the same inputs except for marbling scores, with values recorded either at the fifth rib or at the tenth rib; the predicted outputs are with respect to the aging times of 10 and 20 days, the cooking methods of grill and roast, and the hanging methods of tenderstretch and Achilles tendon.

Overall, the MQ4 scores and the MSA index for 164 carcasses were predicted in total due to some missing data. A precise description and visual support of the MSA methodology and the prediction of MQ4 (MQ4 scores and MSA Index) are indicated in the study of McGilchrist et al. (6), Polkinghorne et al. (14), and Bonny et al. (20).

### Statistical Analysis

All statistical analyses were performed by using the R software (version 3.5.2). Significant differences between means of raw data, MQ4 scores, and the MSA index for the two grading sites were determined by using an ANOVA with “aov” function.

A Pearson's correlation analysis of raw data, MQ4 scores, and the MSA index were performed by using “stat_cor” and “pairs. panels” functions to determine correlation coefficients between the carcass characteristics at both the sites (fifth and tenth ribs).

Linear regression models for MQ4 scores using two sets of marbling scores from the fifth rib and the tenth rib were done by using “lm” (linear model) function.

Scatter plots were made by using “ggscatter” function with “add reg.line” and “stat_cor”.

## Results

### Grading Scores, Predicted MQ4 Scores, and MSA Index

Grading scores of the AUS-MEAT marbling, MSA marbling, meat color, and fat color which were assessed on the LT muscle at the fifth rib and the tenth rib are presented in Table 2. There were no significant differences between the values of AUS-MEAT and the MSA marbling scores at two different locations. In contrast to marbling, there were significant differences in the meat and the fat color between the fifth rib and the tenth rib. The meat color at the tenth rib was significantly darker than that at the fifth rib (*p* < 0.01). The fat color at the tenth rib was significantly yellower than that at the fifth rib (*p* < 0.001) (Table 2).

**Table 2:** Number, mean, minimum, maximum scores, SEM of marbling, meat color, and fat color scores determined at the fifth and the tenth ribs.

	**5^th^ rib**				**10^th^ rib**					
	***n***	**Mean**	**Min**	**Max**	***n***	**Mean**	**Min**	**Max**	**SEM**	***P*-value**
AUS MB^1^	208	0.7	0	5	207	0.7	0	4	0.06	0.95
MSA MB^2^	208	288	100	750	207	291	100	680	7.2	0.73
Meat color	208	2.5^b^	1B	6	208	2.9^a^	1B	7	0.07	0.003
Fat color	197	2.5^b^	0	6	196	3.5^a^	0	9	0.11	<0.001
MQ4 CUB045^3^	164	58	46	72	164	58	46	69	0.39	0.97
MQ4 STA045^4^	164	52	40	67	164	52	40	65	0.42	0.95
MQ4 STP045^5^	164	50	37	66	164	50	37	63	0.44	0.94
MQ4 OYS036^6^	164	64	57	73	164	64	57	72	0.27	0.94
MQ4 BLD096^7^	164	48	37	61	164	48	37	58	0.39	0.98
MQ4 RMP131^8^	164	46	36	58	164	46	36	57	0.39	0.92
MQ4 KNU066^9^	164	41	31	53	164	41	31	51	0.37	0.97
MQ4 OUT005^10^	164	38	28	49	164	38	28	49	0.39	0.95
MQ4 EYE075^11^	164	37	25	54	164	37	25	51	0.47	0.99
MQ4 CHK074^12^	164	55	45	67	164	55	45	65	0.34	0.99
MSA index	164	52	43	64	164	52	42	62	0.38	0.92

1 *AUSMB, AUS-MEAT Marbling score*;

2 *Meat Standards Australia (MSA) MB, MSA Marbling score*;

3 *Meat quality CUB045, MQ4 score of CUB045 (M. longissimus thoracis)*;

4 *MQ4 STA045, MQ4 score of STA045 (M. longissimus thoracis et lumborum, anterior striploin piece)*;

5 *MQ4 STP045, MQ4 score of STP045 (M. longissimus thoracis et lumborum, posterior striploin piece)*;

6 *MQ4 OYS036, M. infraspinatus*;

7 *MQ4 BLD096, M. triceps brachii caput longum*;

8 *MQ4 RMP131, M. gluteus medius*;

9 *MQ4 KNU066, M. rectus femoris*;

10 *MQ4 OUT005, M. biceps femoris*;

11 MQ4 EYE075, M. semitendinosus; and

12 *MQ4 CHK074, M. semispinalis capitis; hang method: AT (Achilles tendon), aging time: 10 days, cooking method: grill*.

a,b *Within a row, means with different letters are significantly different (p < 0.05) between the fifth and the tenth ribs*.

In addition to the marbling scores, no significant difference was observed between the fifth rib and the tenth rib for the predicted MQ4 scores of three muscle cuts, namely *M*. LT, *M*. LTL at the anterior striploin piece, and *M*. LTL at the posterior striploin piece (Table 2). Similarly, no significant difference was observed for the predicted MSA scores for other cuts (Table 2), as well as for the MSA index calculated from the MQ4 of the different cuts of the carcass (Table 2).

### Correlations Between Carcass Characteristics and Grading Scores at the Two Grading Sites

Table 3 presents the correlations between the ossification score (which reflects animal maturity) and the ribeye assessment scores (AUS-MEAT marbling, MSA marbling, meat color, and fat color) either at the fifth rib or at the tenth rib.

**Table 3:** Correlation coefficients (*r*) among the assessment scores at the fifth rib and the tenth rib.

	**AUSMB^1^ 10^th^**	**MSAMB^2^ 5^th^**	**MSAMB 10^th^**	**MC^3^ 5^th^**	**MC 10^th^**	**FC^4^ 5^th^**	**FC 10^th^**	**OSS^5^**
AUSMB 5^th^	0.77^***^	0.88^***^	0.74^***^	0.06	0.16^*^	−0.1	−0.2^**^	0.21
AUSMB 10^th^	1	0.75^***^	0.91^***^	0.09	0.13	−0.14	−0.2^**^	0.18
MSAMB 5^th^		1	0.79^***^	0.02	0.21^***^	−0.14	−0.19^**^	0.31^*^
MSAMB 10^th^			1	0.06	0.21^***^	−0.15^*^	−0.19^**^	0.18
MC 5^th^				1	0.43^***^	0.1	0.06	0.1
MC 10^th^					1	0.1	0.13	0.03
FC 5^th^						1	0.7^***^	0.39^***^
FC 10^th^							1	0.36^***^

1 *AUSMB, AUS-MEAT Marbling*;

2 *MSAMB, MSA Marbling*;

3 *MC, Meat Color*;

4 FC, Fat Color; and

5 *OSS, Ossification*.

***, **, * *indicate that correlation is significantly different at the 0.001 level (p < 0.001), 0.01 level (p < 0.01), and 0.05 level (p < 0.05)*.

Strong relationships for the marbling score were observed either for AUS-MEAT measurements and MSA measurements. Indeed, the correlation coefficients between the fifth rib and the tenth rib range from 0.74 to 0.91 (*p* < 0.001).

Furthermore, there was a moderate correlation for the meat color between the fifth rib and the tenth rib (*r* = 0.43, *p* < 0.001). A significant correlation for fat color between the fifth rib and the tenth rib was also observed (*r* = 0.70, *p* < 0.001). In addition, the ossification score had a significant and positive correlation with fat color scores at both the fifth and the tenth ribs (*r* = 0.39, *r* = 0.36, *p* < 0.001). However, ossification was not significantly associated with meat color.

### Relationships of the MSA Index and MQ4 Scores Predicted From Marbling Scores Between the Two Grading Sites

**Figure 1:**
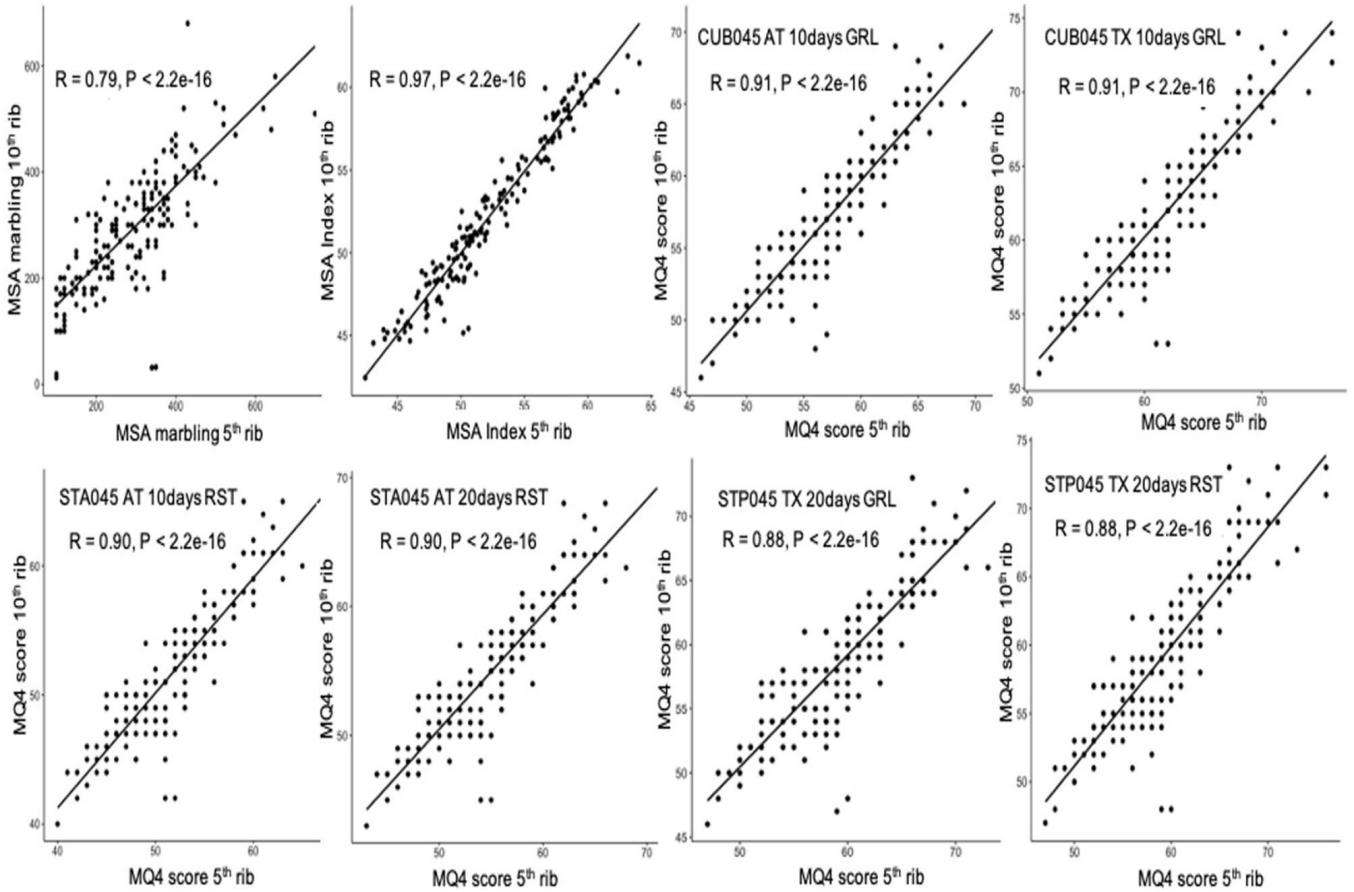
Scatterplots of the Meat Standards Australia (MSA) index and meat quality (MQ4) scores with inputs of marbling scores from the fifth rib (x-axis) or the tenth rib (y-axis). CUB045, *M. longissimus thoracis*; STA045, *M. longissimus thoracis et lumborum*, anterior striploin piece; STP045, *M. longissimus thoracis et lumborum*, posterior striploin piece; AT, hang method—Achilles tendon; TX, hang method: tenderstretch; 10 days, 10 days aging; 20 days, 20 days aging; GRL, cooking method—grill; and RST, cooking method—roast.

For further implementation of the MSA grading scheme based on the grading at the fifth rib, it is crucial to determine the strength of the relationships between the MQ4 scores predicted by using the marbling score at the fifth rib or at the tenth rib. The results of the correlation analyses between the MQ4 scores for 10 cuts with 4 output combinations of the cooking method with hanging method, and with aging time are presented in Table 4. In addition, very small differences in the MQ4 scores between the fifth rib and the tenth rib can be observed as shown in Table 4. Also, the results of correlation analyses for the MSA index and the MQ4 score mainly for the three LT muscles are shown in scatter plots (Figure 1). Correlation coefficients were very high (from 0.88 to 0.98) and almost similar among the different output groups. For OUT005, extremely strong correlations (*r* = 0.98, *p* < 0.001) between the MQ4 scores were observed for the four groups. For CUB045, STA045, and STP045, strong and similar correlations between the MQ4 scores were observed by using the marbling inputs from either the fifth rib or the tenth rib, and with different aging times or hanging methods, the similar distribution and strong correlation for the MSA index and the MQ4 scores between the two grading sites can also be seen in Figure 1. Similarly, significantly strong relationships were observed for the MQ4 scores between the two grading sites for all other cuts with different output combinations, and the distribution and correlation are assumed to be the same strong as the three LT muscles. In addition, whereas the correlations between the two sets of MSA marbling scores was 0.79 (*p* < 0.001), there was a very significant strong correlation between the MSA indexes predicted from the marbling score of the fifth rib and the tenth rib (*r* = 0.97, *p* < 0.001) (Figure 1).

**Table 4:** Pearson correlations and average differences between the MQ4 scores predicted by the marbling score assessed at the fifth rib and the tenth rib.

**Cut**	**Cooking method: Grill**				**Cooking method: Roast**			
	**Hang method: Achilles Tendon**				**Aging time: 10 days**			
	**Aging time**				**Hang method**			
	**10 days**		**20 days**		**Achilles Tendon**		**Tenderstretch**	
	**Coefficient**	**Average difference (SD)**	**Coefficient**	**Average difference (SD)**	**Coefficient**	**Average difference (SD)**	**Coefficient**	**Average difference (SD)**
CUB045^1^	0.91^***^	0.03 (2.05)	0.91^***^	0.03 (2.05)	0.91^***^	0.03 (2.05)	0.91^***^	0.03 (2.05)
STA045^2^	0.89^***^	0.07 (2.44)	0.89^***^	0.07 (2.44)	0.90^***^	0.07 (2.44)	0.89^***^	0.07 (2.44)
STP045^3^	0.88^***^	0.07 (2.67)	0.88^***^	0.07 (2.67)	0.89^***^	0.07 (2.67)	0.89^***^	0.07 (2.67)
OYS036^4^	0.97^***^	0.04 (0.77)	0.97^***^	0.04 (0.77)	0.97^***^	0.04 (0.77)	0.97^***^	0.04 (0.77)
BLD096^5^	0.97^***^	0.04 (1.15)	0.97^***^	0.04 (1.15)	0.97^***^	0.04 (1.15)	0.97^***^	0.04 (1.15)
RMP131^6^	0.98^***^	0.02 (0.76)	0.99^***^	0.02 (0.76)	0.99^***^	0.02 (0.76)	0.98^***^	0.02 (0.76)
KNU066^7^	0.97^***^	0.02 (1.14)	0.97^***^	0.02 (1.14)	0.97^***^	0.02 (1.14)	0.97^***^	0.02 (1.14)
OUT005^8^	0.98^***^	0.01 (0.98)	0.98^***^	0.01 (0.98)	0.98^***^	0.01 (0.98)	0.98^***^	0.01 (0.98)
EYE075^9^	0.98^***^	0.02 (1.14)	0.98^***^	0.02 (1.14)	0.98^***^	0.02 (1.14)	0.98^***^	0.02 (1.14)
CHK074^10^	0.94^***^	0.03 (1.52)	0.94^***^	0.03 (1.52)	0.93^***^	0.03 (1.52)	0.93^***^	0.03 (1.52)

1 *CUB045, M. longissimus thoracis*;

2 *STA045, M. longissimus thoracis et lumborum. anterior striploin piece*;

3 *STP045, M. longissimus thoracis et lumborum. posterior striploin piece*;

4 *OYS036, M. infraspinatus*;

5 *BLD096, M. triceps brachii caput longum*;

6 *RMP131, M. gluteus medius*;

7 *KNU066, M. rectus femoris*;

8 *OUT005, M. biceps femoris*;

9 EYE075, M. semitendinosus; and

10 *CHK074, M. semispinalis capitis*.

*** *p < 0.001*.

*MQ4 scores are indicated on a scale from 0 to 100*.

Linear regression was used to determine the contribution of the marbling score either from the fifth rib or from the tenth rib to explain the variability in the predicted MQ4 score. As shown in Table 5, for one individual cut, the MQ4 scores for the two models regressed by using the marbling score at the two grading sites are almost similar. For STA045 and STP045, the coefficients of determination (*R*^2^ = 0.45, *R*^2^ = 0.50) are relatively higher in comparison to other cuts, which are understandable since the marbling assessment was carried out on the *M*. LT. By contrast, a much lower *R*^2^-value can be found for the other cuts, such as RMP131 and EYE 075 (*R*^2^ = 0.02).

**Table 5:** Linear regression equations for indicating the MQ4 score using the marbling score assessment at the fifth rib and the tenth rib.

**Cut**	**5^th^ rib**	**10^th^ rib**
CUB045^1^	MQ4 score = 0.024^***^marbling+50.32 (*R*^2^ = 0.37)	MQ4 score = 0.026^***^marbling+50.02 (*R*^2^ = 0.37)
STA045^2^	MQ4 score = 0.028^***^marbling+43.08 (*R*^2^ = 0.45)	MQ4 score = 0.031^***^marbling+42.51 (*R*^2^ = 0.47)
STP045^3^	MQ4 score = 0.032^***^marbling+39.95 (*R*^2^ = 0.50)	MQ4 score = 0.034^***^marbling+39.55 (*R*^2^ = 0.51)
OYS036^4^	MQ4 score = 0.012^***^marbling+60.43 (*R*^2^ = 0.19)	MQ4 score = 0.014^***^marbling+60.05 (*R*^2^ = 0.22)
BLD096^5^	MQ4 score = 0.011^***^marbling+44.36 (*R*^2^ = 0.07)	MQ4 score = 0.012^***^marbling+44.24 (*R*^2^ = 0.07)
RMP131^6^	MQ4 score = 0.006^***^marbling+43.62 (*R*^2^ = 0.02)	MQ4 score = 0.007^***^marbling+43.47 (*R*^2^ = 0.03)
KUN066^7^	MQ4 score = 0.011^***^marbling+37.25 (*R*^2^ = 0.08)	MQ4 score = 0.012^***^marbling+37.14 (*R*^2^ = 0.09)
OUT005^8^	MQ4 score = 0.009^***^marbling+34.67 (*R*^2^ = 0.05)	MQ4 score = 0.010^***^marbling+34.56 (*R*^2^ = 0.06)
EYE075^9^	MQ4 score = 0.006^***^marbling+34.63 (*R*^2^ = 0.02)	MQ4 score = 0.006^***^marbling+34.87 (*R*^2^ = 0.01)
CHK074^10^	MQ4 score = 0.020^***^marbling+48.61 (*R*^2^ = 0.30)	MQ4 score = 0.022^***^marbling+48.12 (*R*^2^ = 0.34)

1 *CUB045, M. longissimus thoracis*;

2 *STA045, M. longissimus thoracis et lumborum, anterior striploin piece*;

3 *STP045, M. longissimus thoracis et lumborum, posterior striploin piece*;

4 *OYS036, M. infraspinatus*;

5 *BLD096, M. triceps brachii caput longum*;

6 *RMP131, M. gluteus medius*;

7 *KNU066, M. rectus femoris*;

8 *OUT005, M. biceps femoris*;

9 EYE075, M. semitendinosus; and

10 *CHK074, M. semispinalis capitis*.

*** *p < 0.001*.

## Discussion

### Marbling Scores at the Fifth and the Tenth Ribs

The marbling score was developed to estimate the intramuscular fat in the ABCAS system to be used in the MSA grading scheme with the ultimate objective to ensure the eating quality at the consumer end (20). In 2018, within the framework of the French national food conference (8), the French meat sector represented by Interbev recommended introducing the marbling score into the French beef grading scheme. As a result, the marbling assessment has been gradually introduced in one of the French local meat plants for the premium beef brand called “Or Rouge” based on the late-maturing Limousine breed. In this study, no significant difference in the marbling score was observed between the fifth rib and the tenth rib sites. In addition, the marbling scores assessed at these two sites were quite equally distributed up to 400, and higher levels were also observed. The level of marbling in French cattle is typically much lower than that in Australia (21), but the marbling level of the current sample is not very low and the marbling level between the two studied sites is indeed similar. In addition, we observed a strong relationship between the AUS-MEAT marbling score and the MSA marbling score, which suggests a strong consistency of the marbling score between the AUS-MEAT measurement and the MSA measurement [*r* = 0.88 (between MSAMB and AUSMB at the fifth rib), *r* = 0.91 (between MSAMB and AUSMB at the tenth rib)], as well as between the fifth rib and the tenth rib [*r* = 0.77 (between the AUSMB scores at the fifth and tenth ribs), *r* = 0.79 (between the MSAMB scores at the fifth and tenth ribs)]. These findings are consistent with that of Kruk et al. (22), who reported a high association between the AUS-MEAT marbling score and the MSA marbling score (*r* = 0.76). Similar to our results, Cook et al. (23) also found that the marbling scores from the thirteenth thoracic vertebrae to the fifth lumbar vertebrae were similar. Taylor and Johnson (24) observed that the intramuscular fat content at the fifth rib was slightly higher than that at the tenth rib, whereas the marbling scores between the fifth rib and the tenth rib were almost the same. In addition, it was found that the marbling score of *M*. LT highly correlated with that of *M. longissimus lumborum* (LL) (*r* = 0.83) (25).

Our findings of marbling consistency between the different ribs were obtained by using the grader assessment. Indeed, when the graders score the carcasses, a small difference in marbling has been found across the different muscles and especially between STR045 and CUB045, in comparison with near infrared spectroscopy (NIR) measurements (25). Indeed, recent developments in the marbling assessment tend to use instrument-grading systems, which are likely to be more precise in comparison to human carcass graders. With this new type of technology, Acheson et al. (26) observed that the marbling score decreased from the thirteenth thoracic vertebrae to the fifth lumbar vertebrae. The contradictory results from the human graders may be due to the different marbling grading processes. In study by Acheson et al., a computer vision system, cold camera, and proprietary software were used to assess marbling, which seems to be a more objective and repeatable method in comparison to the assessment provided by the carcass grader. Nonetheless, Schulz and Sundrum (27) observed that the marbling scores at the tenth, eleventh, twelfth, and thirteenth ribs were strongly correlated (*r* = 0.80–0.89) by using a camera grading technology.

### Marbling Is One of the Most Important Traits for Beef Eating Quality

Beef quality has a multifactorial determinism as shown by the various inputs in the MSA model (i.e., ossification and marbling scores, cut, aging time, hanging method, cooking method, and even use of hormone growth promoters). However, as most of these factors are fixed in this study (one aging time, one hanging method, and a limited range in the ossification score), variability in the marbling score becomes an important trait, which significantly contributes to beef eating quality.

In Europe, the main factors of carcass grading are the European conformation score and the fat score, which are compulsory (shown in Table 1) and therefore routinely used by the European beef industry. However, there is very poor and/or no relationship between the European classification scores and marbling, as well as with beef eating quality (28, 29). By contrast, marbling is not widely measured in Europe, particularly in France, except in some cases of “Label Rouge” animals (Label Rouge is officially a national label for food, non-food, and unprocessed agriculture products in France). In the slaughterhouse where this work was undertaken, the measurement of marbling was done for the beef premium brand of the Beauvallet Company “Or Rouge” based on the Limousine breed. Furthermore, high-marbled beef does not seem to be welcomed by the French consumers. Indeed, according to a beef consumption survey conducted in France (4), the low willingness to purchase beef for a quarter of the respondents is mainly due to their concerns of health risks caused by excess fat content, but marbling does contribute to eating quality. However, the willingness to purchase beef among French consumers decreased from 70% (before tasting) to 55% (after tasting) when beef samples were low-marbled the willingness to purchase beef among the same group of consumers increased from 30% (before tasting) to 80% (after tasting) when beef samples were high- marbled (30). This indicates that, even if a highly visible fatty meat seems unacceptable, the better eating quality of high-marbled beef could meet their eating expectations.

Most of the American and Australian consumers also prefer the visual appearance of low-marbled beef (31, 32). However, with efforts to increase the popularization of the relationships between intramuscular fat and eating quality, consumers started to change their mind and embrace fattier meat and even high-marbled beef (33). Consumers from Asian countries, particularly in Japan, are well-known to enjoy high-marbled beef, but Japanese consumers do also enjoy moderately marbled beef (34). Although the preferences of consumers for marbling levels differ across various countries, marbling is undoubtedly one of the multiple traits that highly contribute to beef eating quality (35), but its contribution to eating quality seems to vary according to the muscle as predicted by this study (Table 5).

Intramuscular fat deposition, and therefore marbling, depends on many factors such as nutrition, genetics, and, to a lower extent, animal maturity (36, 37). However, no correlation or a weak correlation (only with the MSA marbling score at the fifth rib) was found between the ossification score and the MSA marbling score (Table 2). This may be due to the carcasses graded in this study, which were from old animals (cows) of the Limousine breed and of similar age. This breed is a late maturing one and, more importantly, produces low-marbled beef (38). However, even the late-maturing cows develop more marbling than younger counterparts as they become older and physiologically mature. In fact, the marbling level is influenced by various factors such as expression and the presence of cellular factors (39). The processes determining the development of marbling of mature cows are poorly studied and require further investigation. The factors such as genetics, whole body fatness, energy intake previous to slaughter, and lifetime fat turnover associated with raising calves are potential subjects for future research.

### Meat Color Characteristics at the Fifth Rib and the Tenth Rib

From a retail point of purchase, the meat color is one of the most critical traits for consumers to purchase beef (40). Various factors, such as diet, pH, and muscle type, and characteristics affect the meat color (41). The meat color depends on the ultimate pH that gradually increases from lumbar to thoracis, the pH at the fourth rib (LT) being higher than at the eleventh rib (LL) (42). Accordingly, LT should be darker than at the *lumborum* vertebrae (43). Contrary to this assumption and the previous observations in Australia with other animal types (15), the present study showed that the muscle at the tenth rib (LL) was significantly darker than the muscle at the fifth rib, suggesting the involvement of other factors.

The meat color also partly depends in part on the muscle fiber type (44). Indeed, oxygen diffusion is related to the muscle fiber type and results in more or less oxymyoglobin (45). The proportions of type I and type II A fibers in LT are higher than those in LL, the proportion of type II B fiber in LL is higher than that in LT (46). However, oxidative fibers (I and IIA) are known to have a decreased rate in the extent of postmortem pH decline and lightness and inherently have an increase in redness due to a higher myoglobin concentration, thus resulting in darker meat when compared to glycolytic muscles (type II B) (47). In this way, the LT muscle is expected to be darker than the LL muscle. The current finding indicates that the meat color of LT at the tenth rib was darker than that at the fifth rib. Even though the tenth rib is close to the *lumborum* vertebrae, the muscle on the fifth rib and the tenth rib is still the LL muscle. The fiber type is therefore unlikely to be a reason for the observed color difference.

Practically, the meat color is not used in the current MSA grading system, but with it being used as a threshold in the old MSA system, the meat color should be three or less than three, and the carcass with a meat color more than four had to be rejected by the system. The proportion of the meat color higher than four at the fifth rib and the tenth rib is calculated in this study, and is 18% and 29%, respectively. In this way, the meat color assessment conducted at the tenth rib would have increased the possibility of being rejected in the older MSA system for some of the carcasses.

### Fat Color Characteristics at the Fifth Rib and the Tenth Rib

The fat color is of practical importance for the beef industry since purchase willingness of the consumer is affected by the color, the white fat color being more desirable than the yellow fat color in many countries (48, 49). In the European market, too much yellow fat on the carcass is considered unacceptable (50). The fat color depends on age, gender, genotype, and nutrition. The yellowness is mainly explained by carotenes accumulating in fat tissue (48).

Whereas, Meat Livestock Australia indicates that carcasses may be ribbed at any site between the fifth rib and the thirteenth rib for grading (15), in the current study, the fat color score (3.5) at the tenth rib was significantly higher (and therefore yellower) than that at the fifth rib (2.5). Acheson et al. (26) reported that animals deposit intramuscular fat from the anterior to the posterior along the vertebrae. It is, therefore, possible to speculate that the significant difference in fat color between the fifth rib and tenth rib may also be due to a different accumulation rate of carotenoids between the posterior and anterior intermuscular fat. With an increase in maturity, more carotenoids could be concentrated at the tenth rib than that at the fifth rib. The significantly positive correlation observed between the fat color and the ossification score may also support the above hypothesis. This result supports the evidence that adipose tissues become more yellow as animal maturity increases (51). Moreover, Moon et al. (52) indicated that high-marbled beef tended to have a lower yellowness fat color due to the dilution of pigments in more fat, which is in line with a significantly negative correlation between fat color and marbling (Table 3).

### MQ4 Scores and the MSA Index at the Fifth Rib and the Tenth Rib

The MSA beef eating quality score (MQ4) is a combination score of tenderness, juiciness, flavor liking, and overall liking for the individual cuts with a defined hanging method, aging time, and the cooking method (14). Marbling, as one of the input parameters, is used to predict the eating quality score (MQ4) of the individual cuts in the MSA cut-based model (20). The MSA index is a global score of the average eating quality and the potential merit of a whole beef carcass, which is calculated from the predictive eating quality scores of 39 MSA cuts. To further confirm the feasibility that the marbling assessment could be conducted at the fifth rib and not influence the eating quality prediction of individual cuts and the whole carcass, correlation analyses were performed with ten cuts to evaluate the relationships between the MQ4 scores at the fifth rib and the tenth rib. The results in the correlation analyses and scatter plots evidenced high and significant correlation coefficients and a similar distribution of the scores, demonstrating that the marbling assessment at either the fifth rib or the tenth rib has an extremely low impact on the prediction of the MQ4 scores for each cut. In addition, similar and strong correlations between the MQ4 scores predicted from the marbling scores recorded at the two ribs were observed for different hanging methods, aging times, and cooking methods. Moreover, the two MQ4 score models regressed by using the marbling score for one specific cut were found highly similar between the two marbling assessment sites (Table 5). Furthermore, we observed the same mean value of the MSA index (52) with no statistical difference (*p* = 0.92, Table 2) and significantly strong correlations between the MSA indexes predicted from the marbling scores from the fifth rib and the tenth rib (*r* = 0.97, *p* < 0.001, data not shown). All of these results indicate that the marbling assessment at the fifth rib or the tenth rib has no or very little impact on the prediction of the MQ4 score and the MSA index for different production and process combinations.

Another interesting finding is that the correlation coefficients between the MQ4 scores of the two grading sites was extremely high for some cuts, such as OUT005, i.e., higher than STA045 and STP045, while the regression model of OUT005 has a very low explanatory power (*R*^2^-value) when using the marbling score to explain the variability of the MQ4 score. This is in-line with the fact that the marbling score measured on *M*. LTL might have less impact on the prediction of the MQ4 scores for other cuts due to a low or moderate correlation of the marbling scores between different cuts (25). In summary, without considering the influence of other MSA predictive parameters on the prediction of the MSA index and the MQ4 scores, it is feasible to assess the marbling at the fifth rib to routinely predict the MSA index and the MQ4 scores for French cattle.

## Conclusion

This study has shown that there is no difference in the marbling scores determined by using the accredited trained graders according to the ABCAS protocol, between the fifth rib and the tenth rib, as well as in the predicted MSA index and the MQ4 scores from these two sets of values. This confirms that the marbling score could be determined at the fifth rib by using the accredited trained graders, i.e., where carcasses are generally quartered in Europe. In contrast, meat color and fat color, not taken into the MSA model to predict eating quality of beef, are significantly different between the two grading sites. Given that Limousine cows are an important source of beef in France and that a little detailed work regarding the marbling distribution has been undertaken using cull cows, the current study is considered to be relevant to provide practical recommendations to the European (and especially the French) beef industry. Thus, this work supports potential implications in favor of the MSA implementation for the late maturing and low-marbled cattle breeds, such as Limousine, and also for potential MSA implementation in French beef plants. However, further work is needed to completely study the implementation of the ABCAS carcass grading system with respect to other critical carcass factors according to the MSA methodology while following the European carcass quartering practices.

## Data Availability Statement

The raw data supporting the conclusions of this article will be made available by the authors, without undue reservation.

## Ethics Statement

Ethical review and approval was not required for the animal study because Data from cows was used from a commercial slaughterhouse at Limoges, France according to normal rules.

## Author Contributions

JL performed a statistical analysis and wrote the manuscript. GP and AN provided the data. IL and M-PE-O made a high contribution to the structure of the paper. DP provided prediction of the MQ4 score. J-FH conceived the study. All authors contributed to the article and approved the submitted version.

## Conflict of Interest

The authors declare that the research was conducted in the absence of any commercial or financial relationships that could be construed as a potential conflict of interest.

## References

[1] Hocquette JF Ellies-Oury MP Lherm M Pineau C Deblitz C Farmer L. Current situation and future prospects for beef production in Europe—a review. Asian-australas. J Anim Sci. (2018) 31:1017. 10.5713/ajas.18.0196 PMC6039334 29807416

[2] Sans P Legrand I. Tendance d'évolution des caractéristiques des marchés. In: Ellies-Oury MP Hocquette JF, editors. La Chaîne de La Viande Bovine. Production, transformation, valorisation et consommation. Paris: Editions Lavoisier (2018). p. 125–42.

[3] FranceAgriMer. Consommation des produits carnés en 2014, 4, Montreuil. (2015). p. 15.

[4] Ellies-Oury MP Lee A Jacob H Hocquette JF. Meat consumption–what French consumers feel about the quality of beef? Ital. J Anim Sci. (2019) 18:646–56. 10.1080/1828051X.2018.1551072

[5] Meat and Livestock Australia (2010). Available online at: https://www.mla.com.au/globalassets/mla-corporate/generic/about-mla/annual-report-2009_10.pdf (accessed October 30, 2010).

[6] McGilchrist P Polkinghorne RJ Ball AJ Thompson J. The meat standards Australia index indicates beef carcass quality. Animal. (2019) 13:1750–7. 10.1017/S1751731118003713 30724139 PMC6639719

[7] Institut de l'Elevage. Les chiffres clés du GEB. Bovins 2020. Productions lait et viande. (2020). Available online at: http://idele.fr/no_cache/recherche/publication/idelesolr/recommends/chiffres-cles-bovins-2020.html (accessed September 23, 2020).

[8] Etats Généraux de l'Alimentation EGA (2018). Available online at: https://agriculture.gouv.fr/alimagri-les-etats-generaux-de-lalimentation (accessed October 2, 2018).

[9] Meat and Livestock Australia (2011). Available online at: https://www.mla.com.au/globalassets/mla-corporate/generic/about-mla/anual-report-2010-11-final.pdf (accessed October 30, 2011).

[10] Legrand I Hocquette JF Polkinghorne RJ Pethick DW. Prediction of beef. eating quality in France using the Meat Standards Australia system. Animal. (2013) 7:524–9. 10.1017/S1751731112001553 23031268

[11] Chong FS Farmer LJ Hagan TDJ Speers JS Sanderson DW Devlin DJ. Regional, socioeconomic and behavioural-impacts on consumer acceptability of beef in Northern Ireland, Republic of Ireland and Great Britain. Meat Sci. (2019) 154:86–95. 10.1016/j.meatsci.2019.04.009 31022586

[12] Pogorzelski G Wozniak K Polkinghorne RJ Wierzbicka A Półtorak A. Polish consumer categorisation of grilled beef at 6mm and 25mm thickness into quality grades, based on Meat Standards Australia methodology. Meat Sci. (2020) 161:1–7. 10.1016/j.meatsci.2019.107953 31675648

[13] Hocquette JF Ellies-Oury MP Legrand I Pethick D Gardner G Wierzbicki J. Research in beef tenderness and palatability in the era of big data. Meat Muscle Biol. (2020) 4:1–13. 10.22175/mmb.9488

[14] Polkinghorne R Thompson JM Watson R Gee A Porter M. Evolution of the Meat Standards Australia (MSA) beef grading system. Austral J Exp Agric. (2008) 48:1351–9. 10.1071/EA07177

[15] AUS-MEAT. Australian Beef Carcase Evaluation Chiller Assessment. Version 9, 16/07/2018. Murarri, QLD: AUS-MEAT Limited (2018).

[16] Meat Standards Australia beef information kit – MLA Australia livestock (2010). Available online at: https://www.mla.com.au/globalassets/mla-corporate/marketing-beef-and-lamb/msa_tt_beefinfokit_jul13_lr.pdf

[17] Hickey JM Keane MG Kenny DA Cromie AR Veerkamp RF. Genetic. parameters for EUROP carcass traits within different groups of cattle in Ireland. J Anim Sci. (2007) 85:314–21. 10.2527/jas.2006-263 17235018

[18] United States Department of Agriculture. Standards for Grades of Slaughter Cattle and Standards for Grades of Carcass Beef. Agricultural Marketing Services, USDWashington A DC, Government Printing Office. (2017). Available online at: https://www.ams.usda.gov/sites/default/files/media/CarcassBeefStandard.pdf (accessed December 18, 2017).

[19] Ferguson DM. Objective on-line assessment of marbling: a brief review. Aust J Exp Agric. (2004) 44:681–5. 10.1071/EA02161

[20] Bonny SP O'Reilly RA Pethick DW Gardner GE Hocquette JF Pannier L. Update of Meat Standards Australia and the cuts based grading scheme for beef and sheep meat. J Integr Agric. (2018) 17:1641–54. 10.1016/S2095-3119(18)61924-0

[21] Hocquette JF Legrand I Jurie C Pethick DW Micol D. Perception in France of the Australian system for the prediction of beef quality (Meat Standards Australia) with perspectives for the European beef sector. Anim Product Sci. (2011) 51:30–6. 10.1071/AN10045

[22] Kruk ZA Pitchford WS Siebert BD Deland MPB Bottema CDK. Factors affecting estimation of marbling in cattle and the relationship between marbling scores and intramuscular fat. Anim Product Austral. (2002) 24:129–32. Available online at: https://www.researchgate.net/publication/265575133_Factors_affecting_estimation_of_marbling_in_cattle_and_the_relationship_between_marbling_score_and_intramuscular_fat

[23] Cook CF Bray RW Weckel KG. Variations in the quantity and distribution of lipid in the bovine longissimus dorsi. J Anim Sci. (1964) 23:329–31. 10.2527/jas1964.232329x

[24] Taylor DG Johnson ER. Visual marbling score and chemical fat content of. *M. Longissimus* in beef carcasses. Proc Austr Soc Anim Product. (1992) 19:71–3.

[25] Konarska M Kuchida K Tarr G Polkinghorne RJ. Relationships between. marbling measures across principal muscles. Meat Sci. (2017) 123:67–78. 10.1016/j.meatsci.2016.09.005 27639062

[26] Acheson RJ Woerner DR Walenciak CE Colle MJ Bass PD. Distribution of marbling throughout the *M. longissimus* thoracis et Lumborum of beef carcasses using an instrument-grading system. Meat Muscle Biol. (2018) 2:303–8. 10.22175/mmb2018.04.0005

[27] Schulz L Sundrum A. Assessing marbling scores of beef at the 10^th^ rib vs. 12^th^ rib of longissimus thoracis in the slaughter line using camera grading technology in Germany. Meat Sci. (2019) 152:116–20. 10.1016/j.meatsci.2019.02.021 30844621

[28] Bonny SPF Pethick DW Legrand I Wierzbicki J Allen P Farmer LJ. European conformation and fat scores have no relationship with eating quality. Animal. (2016) 10:996–1006. 10.1017/S1751731115002839 26755183

[29] Liu J Chriki S Ellies-Oury MP Legrand I Pogorzelski G Wierzbicki J. European conformation and fat scores of bovine carcasses are not good indicators of marbling. Meat Sci. (2020) 170:108233. 10.1016/j.meatsci.2020.108233 32688221

[30] Normand J. Institut de L'Elevage, Lyon. “En France, quelles pistes pour enrayer. la baisse de consommation de viande bovine ?” PowerPoint. (2017). http://idele.fr/fileadmin/medias/Documents/Perception_du_persille_et_du_conditionnement_sous_vide_-_Colloque_Interbev_2018-02-02.pdf (accessed February 2, 2017).

[31] Egan AF Ferguson DM Thompson JM. Consumer sensory requirements for beef and their implications for the Australian beef industry. Aust J Exp Agric. (2001) 41:855–9. 10.1071/EA00065

[32] Killinger KM Calkins CR Umberger WJ Feuz DM Eskridge KM. Consumer visual preference and value for beef steaks differing in marbling level and color. J Anim Sci. (2004) 82:3288–93. 10.2527/2004.82113288x 15542475

[33] Frank D Joo ST Warner R. Consumer acceptability of intramuscular fat. Korean J Food Sci Anim Resour. (2016) 36:699–708. 10.5851/kosfa.2016.36.6.699 28115880 PMC5243953

[34] Sasaki K Ooi M Nagura N Motoyama M Narita T Oe M. Classification and characterization of Japanese consumers' beef preferences by external preference mapping. J Sci Food Agric. (2017) 97:3453–62. 10.1002/jsfa.8204 28071797

[35] Motoyama M Sasaki K Watanabe A. Wagyu and the factors contributing. to its beef quality: a Japanese industry overview. Meat Sci. (2016) 120:10–8. 10.1016/j.meatsci.2016.04.026 27298198

[36] Pethick DW Harper GS Hocquette JF Wang Y. Marbling biology–what do we know about getting fat into muscle. In: Proceedings of Australian Beef–the Leader. Armidale, NSW. (2006). p. 103–10.

[37] Pflanzer SB de Felício PE. Moisture and fat content, marbling level and color of boneless rib cut from Nellore steers varying in maturity and fatness. Meat Sci. (2011) 87:7–11. 10.1016/j.meatsci.2010.08.009 20855172

[38] Malau-Aduli AEO Edriss MA Siebert BD Bottema CDK Pitchford WS. Breed differences and genetic parameters for melting point, marbling score and fatty acid composition of lot-fed cattle. J Anim Physiol Anim Nutr. (2000) 83:95–105. 10.1046/j.1439-0396.2000.00254.x

[39] Kern SA Pritchard RH Blair AD Scramlin SM Underwood KR. The influence of growth stage on carcass composition and factors associated with marbling development in beef cattle. J Anim Sci. (2014) 92:5275–84. 10.2527/jas.2014-7891 25253804

[40] Smith GC Belk KE Sofos JN Tatum JD Williams SN. Economic implications of improved color stability in beef. In: Decker E Faustman C Lopez-Bote CJ, editors. Antioxidants in Muscle Foods: Nutritional Strategies to Improve Quality. New York, NY: Wiley (2000). p. 397–426.

[41] Mancini RA Hunt M. Current research in meat color. Meat Sci. (2005) 71:100–21. 10.1016/j.meatsci.2005.03.003 22064056

[42] Janz JAM Aalhus JL Dugan MER Price MA. A mapping method for the description of Warner–Bratzler shear force gradients in beef Longissimus thoracis et lumborum and Semitendinosus. Meat Sci. (2006) 72:79–90. 10.1016/j.meatsci.2005.06.009 22061377

[43] Hughes JM Kearney G Warner RD. Improving beef meat colour scores at carcass grading. Anim Product Sci. (2014) 54:422–9. 10.1071/AN1345429549843

[44] Ozawa S Mitsuhashi T Mitsumoto M Matsumoto S Itoh N Itagaki K. The characteristics of muscle fiber types of longissimus thoracis muscle and their influences on the quantity and quality of meat from Japanese Black steers. Meat Sci. (2000) 54:65–70. 10.1016/S0309-1740(99)00072-8 22063713

[45] McKenna DR Mies PD Baird BE Pfeiffer KD Ellebracht JW Savell JW. Biochemical and physical factors affecting discoloration characteristics of 19 bovine muscles. Meat Sci. (2005) 70:665–82. 10.1016/j.meatsci.2005.02.016 22063894

[46] Totland GK Kryvi H. Distribution patterns of muscle fibre types in major muscles of the bull (Bos taurus). Anat Embryol. (1991) 184:441–50. 10.1007/BF01236050 1835822

[47] Wicks J Beline M Gomez JFM Luzardo S Silva SL Gerrard D. Muscle energy metabolism, growth, and meat quality in beef cattle. Agriculture. (2019) 9:195. 10.3390/agriculture9090195

[48] Dunne PG Monahan FJ O'Mara FP Moloney AP. Colour of bovine subcutaneous adipose tissue: a review of contributory factors, associations with carcass and meat quality and its potential utility in authentication of dietary history. Meat Sci. (2009) 81:28–45. 10.1016/j.meatsci.2008.06.013 22063959

[49] Ardeshiri A Rose JM. How Australian consumers value intrinsic and extrinsic. attributes of beef products. Food Qual Preference. (2018) 65:146–63. 10.1016/j.foodqual.2017.10.018

[50] Dunne PG O'Mara FP Monahan FJ Moloney AP. Changes in colour characteristics pigmentation of subcutaneous adipose tissue *M. longissimus* thoracisof heifers fed grass, grass silage or concentrate-based diets. Meat Sci. (2006) 74:231–41. 10.1016/j.meatsci.2006.02.003 22062830

[51] Inoue K Shoji N Honda T Oyama K. Genetic relationships between meat. quality traits and fatty acid composition in Japanese black cattle. Anim Sci J. (2017) 88:11–8. 10.1111/asj.12613 27072484

[52] Moon SS Yang HS Park GB Joo ST. The relationship of physiological. maturity and marbling judged according to Korean grading system to meat quality traits of Hanwoo beef females. Meat Sci. (2006) 74:516–21. 10.1016/j.meatsci.2006.04.027 22063056

